# The Forge AHEAD Center Pilot Plus program: An integrated framework combining pilot funding, training, and community-engaged translational science

**DOI:** 10.1017/cts.2026.10779

**Published:** 2026-06-25

**Authors:** Michael Mugavero, Rikki M. Tanner, Trudi V. Horton, April A. Agne, Robert L. Newton, Denise C. Cornelius, Timothy Turner, Andrea L. Cherrington, Gareth Dutton, Larry R. Hearld, Carrie R. Howell, Tina Kempin Reuter, Maria Pisu, Jeffery Walker, Orlando M. Gutierrez

**Affiliations:** 1 The University of Alabama at Birmingham Heersink School of Medicine, Birmingham, AL, USA; 2 Louisiana State University Pennington Biomedical Research Center, Baton Rouge, LA, USA; 3 The University of Mississippi Medical Center, Jackson, MS, USA; 4 Tuskegee University, Tuskegee, AL, USA; 5 The University of Alabama at Birmingham, Birmingham, AL, USA

**Keywords:** implementation science, translational research, collaborative research, health disparities

## Abstract

The Deep South has the highest prevalence of obesity, diabetes, and hypertension in the United States, with marked disparities by race, socioeconomic status, and rurality. Addressing these disparities requires not only effective interventions, but also a translational science workforce equipped to design, implement, and scale solutions in real-world settings. The Forge AHEAD Center was established to improve cardiometabolic health outcomes across the Deep South by advancing both research and scientific workforce development. As a central component of this mission, the Center has developed the “Pilot Plus” model, an integrated framework that combines pilot funding with structured training in translational science, multidisciplinary team-based mentorship, and embedded community engagement. Early-stage investigators are supported through coordinated access to methodological expertise, including dissemination and implantation science and assessment of social determinants of health, while community stakeholders are incorporated into the research process. By aligning funding, training, and infrastructure within a single program, the Pilot Plus model addresses fragmentation in traditional research development approaches and expands access to critical resources across institutions. This model provides an adaptable framework for strengthening translational research capacity and developing a cadre of demographically varied investigators prepared to advance health outcomes in regions with a high burden of chronic disease

## Introduction

Health disparities in the United States (US) result from historical and contemporary political, social, and economic factors that affect access to healthcare, healthy behaviors, and health outcomes. The Deep South is a cultural and geographic subregion of the US historically characterized by a strong agricultural economy. Alabama (AL), Mississippi (MS), and Louisiana (LA) represent the heart of the region, which has a particularly high prevalence of cardiometabolic risk factors, including obesity, diabetes, and hypertension, with significant disparities by race/ethnicity, sex, socioeconomic status, and rurality [1–14]. Reducing these disparities requires expanding the scientific workforce to better reflect the communities most impacted by cardiometabolic disease. Despite national priorities to expand the translational science workforce, existing pilot funding and training mechanisms are often fragmented, with limited integration of community engagement, methodological support for T3–T4 research, and structured career development. As a result, early-stage investigators (ESIs) frequently lack coordinated infrastructure to develop, implement, and scale interventions in real-world settings. This requires not only intentionally increasing the *number* of investigators dedicated to this work, but also the *demographic distribution* of the investigator pool, as well as training investigators to engage in interdisciplinary, collaborative research that includes non-academic partners to translate contextually appropriate findings into community and system-level change [15].

The Forge AHEAD (Achieving Health Excellence Across the Deep South) Center aims to improve cardiometabolic health across AL, MS, and LA, focusing on populations disproportionately affected, including Black Americans, rural residents, and low-income populations. The Center applies a precision public health approach[16] across the care continuum, emphasizing multilevel intervention (individual to societal) across biological, behavioral, socio-cultural, built environment, and healthcare system domains, consistent with the National Institute on Minority Health and Health Disparities framework [17]. To strengthen impact, this also requires addressing the full continuum of care, including prevention, treatment, and management [18].

The Forge AHEAD Center brings together an interdisciplinary group of scientists from four institutions across three states (the University of Alabama at Birmingham [UAB], Tuskegee University, Pennington Biomedical Research Center [PBRC], and the University of Mississippi Medical Center) with complementary skills and expertise relevant to cardiometabolic disease and health outcomes research. A central component of this effort is the development of the Pilot Plus model, an integrated translational research framework combining training, mentorship, and research support for ESIs. The Center comprises an Administrative Core, Investigator Development Core (IDC), Community Engagement Core (CEC), and three research projects, all administratively managed by an Executive Committee. The IDC directs the Pilot Plus program, which supports ESIs through pilot funding, structured training in T3–T4 translational science [19], team-based mentorship, and community engagement. This manuscript describes the development and implementation of this model as a scalable approach for advancing dissemination and implementation, comparative effectiveness, and translational research.

National Institutes of Health priorities emphasize expanding the health disparities workforce through integrated training, funding, and access to core resources such as dissemination and implementation science, cost-effectiveness, and assessment of SDOH. Since 2021, the Center has developed a coordinated infrastructure integrating these elements to strengthen translational research capacity across the Deep South region. The Pilot Plus model advances translational science training in several ways. First, it integrates pilot funding with required, structured training in T3–T4 research, rather than offering training as an ancillary component. Second, it embeds community stakeholders directly into the scientific review process, positioning community relevance as a core determinant of funding decisions alongside scientific merit. Third, the model incorporates multidisciplinary project management teams that extend beyond traditional mentorship to provide continuous project oversight, facilitate problem-solving, and actively support investigators’ progression to independent funding. Finally, it centralizes access to specialized methodological resources including dissemination and implementation science, social determinants of health assessment, and health economics, thereby reducing gaps in research infrastructure across institutions. Together, these elements constitute a coordinated and potentially generalizable approach to accelerating the development of a varied translational science workforce.

## Program overview

The Forge AHEAD Pilot Plus program operationalizes an integrated model combining pilot funding, structured training, community engagement, and centralized methodological support for T3–T4 research. The program supports annual competitive pilot awards and fosters career development through complementary training activities. The program aims to (1) support ESIs conducting cardiometabolic research; (2) provide training in intervention development, implementation, and dissemination; (3) increase external funding success; (4) promote interdisciplinary collaboration with community partners; and (5) evaluate program effectiveness. This program was built on the models of the Center for Clinical and Translational Science (CCTS, the CTSA hub at UAB) Network Interdisciplinary Pilot Program, which has had notable success in soliciting and managing pilot grants from institutions spanning a large 11-member Partner Network, and the Caregiving Affected Research Early-career Scientists Retention at UAB program which established research development programs delivered to cohorts of ESIs [20].

The IDC provides centralized resources through Translational Design and SDOH Assessment sub-units, ensuring access to advanced expertise across institutions regardless of local infrastructure. The Translational Design Unit advises on study scope and aims, identifying feasible T3–T4 study designs and data sources, selecting implementation science measures, and developing analysis plans with sample size estimation in collaboration with the CCTS Biostatistics, Epidemiology, and Research Design (BERD) unit. Health economics expertise is available to assess cost-effectiveness when needed. The SDOH Assessment Unit provides consultation on theories, frameworks, and measures of SDOH to support ESIs in examining socio-ecological influences on cardiometabolic outcomes, and applies geospatial methods using linked data from participants across AL, MS, and LA to evaluate relationships between built and contextual environments and health outcomes. The Center Communications team supports dissemination by developing public-facing materials for scientific and community audiences, including infographics, posters, and social media content, and by producing ongoing outreach highlighting Center Scholars and their work.

## Pilot funding review process

ESIs from the four core institutions and affiliated CCTS partner institutions are eligible to submit a Letter of Intent for pilot funding. The inclusion of these partner institutions allows for applicants from regional Historically Black Colleges and Universities such as Jackson State and Xavier Universities to apply for funding. Applicants must meet criteria for ESIs, defined as post-doctoral fellows, instructors, Assistant or Associate Professors who have not been a principal investigator on a prior independent NIH grant or equivalent and are within 10 years of their terminal degree. New investigators (defined as those who might not meet criteria for ESI, but were transitioning or expanding their research into T3–T4 translational science in multiple chronic diseases) were also eligible to apply.

The Forge AHEAD Pilot Plus program involves a two-staged solicitation of applications. First, LOIs are solicited by engaging deans, department chairs, and senior faculty at the Center’s academic institutions and affiliated partners. The Center institutions work within their own research departments, the CCTS, Nutrition Obesity Research Centers (UAB and PBRC), Diabetes Research Center (UAB), and Institutional Development Award Networks for Clinical and Translational Research (Louisiana Clinical and Translational Science Center and Mississippi Center for Clinical and Translational Research) to advertise the request for applications. Applications are solicited annually, with the submission deadline at least 3 months prior to the release of funds. Applicants provide a two-page research statement, biosketches, and a statement on how the project addresses a Center theme area and applies a precision public health approach. Applicants can propose cardiometabolic research related to human immunodeficiency virus (HIV; the result of a successful supplement award) or other chronic diseases (e.g., kidney disease) if they are thematically linked to cardiometabolic disease.

A Scientific Review Committee (SRC) and members of the Forge AHEAD Community Advisory Board (CAB) review the LOIs for both scientific merit and community relevance using NIH criteria and assess eligibility, investigator qualifications, competitiveness, community engagement, and potential community and scientific impact (Table 1). In the second phase of solicitation, meritorious applicants are invited to submit full (4-page) research proposals that expand upon project aims, significance, innovation, and approach, in addition to a lay summary to facilitate review by community partners. The proposal is required to present a testable hypothesis with clear delineation of the questions being asked, relevance to the Center’s themes, preliminary data (when available), research approach, and analysis plan, and at least two short term measures of potential benefit to society in line with the Translational Science Benefit Model (TSBM). The TSBM is a framework for evaluating the impact of translational science that advocates for including broader outcomes, such as potential downstream health and societal benefits [21]. For example, providing residents in a rural community with access to an evidence-based diabetes self-management program results in improved health for residents, improvements in patient–clinician communication, and subsequent increased community engagement in healthcare. The applicants are also required to include a budget and justification, which is evaluated for feasibility commensurate with available funds. Importantly, the IDC continues to work with those investigators not invited to submit full applications to sharpen their proposals for alternate internal or extramural funding opportunities, including future Forge AHEAD pilot RFAs.

**Table 1.** Forge AHEAD center pilot plus project review criteria Table 1 long description.

Criterion	Description
Research theme	Applications should clearly describe how the proposed research aligns with the mission of the Center.
Approach	Methods must reflect translational research principles, including implementation/dissemination of effective interventions or program evaluation for efficacy and improvement.
Scientific merit	Evaluated based on NIH criteria: significance, innovation, approach, rigor and reproducibility, and potential for broader implementation or dissemination.
Investigative team	Assessment of the Principal Investigator’s training, demonstrated interest in translational (i.e., T3–T4) research, and potential for independence. Co-investigators are evaluated for necessary expertise and complementary skills.
Environment	The environment must support translational research, including details on academic as well as community or clinical settings relevant to implementation.
Timeline	Applications must justify feasibility of project completion within 1 year or provide rationale for a 2-year project period.
Protection of human subjects	A clear plan for human subjects protections must be provided, including any relevant prior IRB documentation.
Integration with center cores	Proposals must specify which Center Cores will be used and how they will support the project.
Budget	Budget and justification are reviewed for feasibility, appropriateness, and completeness.

Investigators submitting full proposals are required to consult with (1) a member of the CCTS BERD group to ensure scientific rigor in analytic methods and data management; and (2) CAB members in the Forge AHEAD CEC to discuss community needs, assess project impact on the community, and develop partnerships with non-academic entities. In addition, based on recommendations of the SRC and community reviewers, some applicants are advised to meet with one or both of the Translational Design and SDOH sub-units during their full application development process.

### SRC review

Each proposal is reviewed on a predetermined set of criteria by one primary and one secondary reviewer. The reviewers are selected based on expertise, and at least one is external (i.e., not at the Scholar’s institution). Each reviewer provides a written critique in an NIH format addressing each proposal’s strengths and weaknesses.

### CAB review

A distinguishing feature of the Pilot Plus model is the formal integration of community stakeholders into the proposal review process, ensuring that community relevance and potential impact are evaluated alongside scientific rigor and allowing community stakeholders to contribute directly to funding prioritization. Two CAB reviewers are chosen based on the project’s description of the scientific area and the community involved. CAB reviewers provide feedback using a template designed to elicit input on scope, impact, and community relevance.

After both reviews are completed, the program co-directors collect all written critiques and distribute them to the SRC members and CAB Chair. The SRC and CAB Chair then meet to review each of the applications using the criteria outlined in Table 1, with priority assigned in accordance with standard NIH Study Section principles and practices. Based on the mean priority scores, recommendations are made to the Executive Committee, which then makes the final funding determinations. All applicants receive a written summary of the review and are provided the opportunity to meet with an SRC member to discuss proposal critiques. Thus, funded applications can be strengthened prior to the start of the study, and applications not selected for funding have an opportunity to enhance success in future grant submissions.

## “Pilot Plus” curriculum

To strengthen the pilot and feasibility program and set it apart as a unique professional development opportunity designed to train Scholars (pilot awardees) in interdisciplinary, collaborative science, we created a set of components that serve as the “Plus” aspect of our Pilot Plus program (Table 2). These components include monthly seminars, a monthly enrichment series, project management teams for each Scholar, and a focus on community-engaged research. These components are designed to provide value to the Scholars in the areas of research project management, skills development, professional development, and peer support/mentoring. Furthermore, the IDC hosts an annual regional Scholars’ meeting to showcase pilot projects and provides consultations and specialized support through the aforementioned Translational Design and SDOH Assessment sub-units and the Center communications team.

**Table 2.** The pilot plus curriculum: components, structure, and functions Table 2 long description.

Component	Structure/frequency	Key activities	Primary functions	Unique contributions (“Plus”)
Monthly seminars & enrichment series	Monthly; virtual and in-person; cohort-based	Didactic sessions, workshops, work-in-progress presentations	Skills development; methodological training; peer learning	Integrates real-time project application with training; structured peer feedback environment
Project management teams	Quarterly meetings; ongoing engagement	Progress review, barrier identification, problem-solving, grant planning	Project oversight; mentorship; career development	Moves beyond mentorship to **active sponsorship** and structured accountability
Community-engaged research integration	Continuous across project lifecycle	Community advisory board input, proposal review, progress presentations, bidirectional engagement	Ensures relevance; strengthens implementation and dissemination	Embeds community stakeholders in **decision-making and evaluation**, not just participation
Annual scholars meeting	Annual; in-person	Project presentations, networking, skills workshops	Dissemination; professional development; collaboration	Regional cross-institutional integration and visibility
Specialized support units (translational design, SDOH, communications)	On-demand consultation	Study design, SDOH measurement, dissemination materials	Technical support; methodological rigor; communication	Centralizes advanced expertise across institutions

SDOH = social determinants of health.

### Monthly seminars & enrichment series

The Pilot Plus program offers training and enrichment activities built on existing resources within its Partner Network. These activities were developed in collaboration with IDC faculty, Administrative Core faculty and staff, CEC faculty, as well as input from Scholars regarding their specific training needs. The curriculum is delivered to Scholars in the form of monthly seminars and enrichment activities, workshops, and regional symposia including both virtual platforms and face-to-face encounters with the common theme of translational methods to advance ongoing cardiometabolic research. Example topics covered include: (1) use of theoretical frameworks such as the TSBM to assess health and societal impacts; (2) community based participatory research; (3) development and adaptation of interventions for delivery in real-world settings; (4) use of novel experimental or quasi-experimental study designs for intervention evaluation; (5) multi-level measurement of social and structural determinants; (6) dissemination and implementation methods; (7) data sources available to address translational research questions; (8) an overview of unstructured data, natural language processing, and data transformation for electronic medical records; and (9) data management and data analysis (e.g., multilevel models). In addition, the monthly seminars allow each Scholar the opportunity to present a work-in-progress for their pilot project and engage in discussion with their peers and Center faculty regarding project successes and challenges.

### Project management teams

For ongoing engagement and oversight, every Scholar is supported by a multidisciplinary project management team that extends beyond traditional mentorship to provide continuous oversight, accountability, and problem-solving support. The team includes the IDC program director and 3–5 faculty investigators determined most appropriate for the Scholar’s project and professional development needs. These include subject matter experts, research methods representatives from the IDC, the Scholar’s primary mentor(s) and others deemed to be valuable to the successful completion of the pilot project and subsequent research proposals. Faculty at an awardee’s institution are included on project management teams to help trouble-shoot issues and navigate any institutional barriers that might arise. Each team meets at least quarterly during the project period, and is tasked with reviewing progress, identifying barriers, brainstorming solutions, and offering guidance about the successful completion of the project.

The project management team also serves as an active sponsorship role for the Scholars, providing strategic guidance on grant development, career trajectory planning, and positioning for subsequent extramural grant funding. A portion of each project management team meeting is devoted to discussion of the Scholar’s plans for dissemination of the current pilot findings to both scientific and community audiences, as well as plans and timeline for upcoming grant proposals. Members of the project management team provide detailed input for each Scholar regarding appropriate funding mechanisms, timelines for submissions, and overall research career trajectory plans. Over 75% of Scholars have requested the meetings continue beyond the original period of the pilot project, suggesting that this role has been particularly valuable.

### Focus on community-engaged research

Scholars are expected to incorporate community engagement throughout all phases of their research, from proposal development to dissemination, with structured opportunities for bidirectional input from community partners. Through a close relationship with the CEC, Scholars receive regular exposure to community-engaged research topics through monthly seminars, annual in-person meetings that include CAB members and community partners, and regular presentations to the CAB to review project progress and provide feedback from the community perspective. Additionally, Scholars interact with the CAB on an ad hoc basis for input regarding project-specific issues or guidance on their program of research more broadly.

## Program impact

Since 2021, the Center has funded 4–7 pilots per year with a maximum budget of $50,000 in direct costs per project. Projects are funded for one year, with the possibility of a second year of funding with competitive renewal. To date, the Forge AHEAD Center has awarded 33 pilot awards in a diverse range of topics to 30 investigators across 5 institutions, for a total of $1.7M (Table 3). The investigator base is over 75% female and predominantly (90%) Assistant Professors at the time of proposal submission. Overall, approximately 47% are white (14% of whom are Hispanic), 17% are Asian, 33% are Black, and 3% are other races. A mean of 10 Scholars participated in the monthly Scholars’ seminars and a mean of 9 Scholars participated in the monthly enrichment seminars. A Scholars’ workshop held in conjunction with our annual Center meeting is focused on skills and professional development topics, and a range of 7–14 Scholars have participated in the workshops and accompanying poster sessions over the award period. Together, these Scholars have produced over 200 peer-reviewed publications and nearly 100 scientific presentations during their cumulative affiliation with the Center (across all research activities and funding sources since 2021). As of April 2026, 11 publications are directly attributable to Forge AHEAD-funded pilot projects, with additional pilot awards still in progress. Pilot awards are staggered in initiation and completion, with over 50% funded in the past 2 years. Scholarly outputs are expected to accrue over time beyond the active funding period. In addition to tracking scholarly productivity, the IDC is collecting data on extramural grant submissions, funding outcomes, and mechanisms pursued by Scholars. However, given the relatively recent implementation of the program, these outcomes are not yet fully available for analysis. Future evaluations will assess these metrics as key indicators of program effectiveness in advancing investigators to independent funding. The Core also evaluates short-term health and societal impact using the TSBM (Table 4) [21]. Finally, the IDC conducts follow-up surveys of pilot awardees to assess outcomes in terms of career advancement, engagement in teams, as well as to assess the disciplinary impact of projects using bibliometric analysis and other innovative methods. Feedback from each funded investigator is collected and reviewed by the IDC co-directors for opportunities to improve program effectiveness.

**Table 3.** Forge AHEAD center funded pilot projects Table 3 long description.

Thematic area	Project title and funding year	Institution	Target population	Study type / approach
Cardiometabolic risk & clinical interventions	Cardiometabolic Complications in Women with Polycystic Ovarian Syndrome: Role of Androgens, Race/Ethnicity, and the Renin–Angiotensin System (2022)	UMMC	Women with Polycystic Ovarian Syndrome	Observational
	Tracking Outcomes of Hypertension in Women with Breast Cancer (2022)	UMMC	Women with breast cancer and hypertension	Observational/ Outcomes Research
	Improving Patient Knowledge of Heart Failure with Preserved Ejection Fraction: Patient-Centered and Community-Engaged Pilot Study (2022)	UAB	Adults with heart failure with preserved ejection fraction	Behavioral/Education Intervention
	Post-Emergency Department Discharge Clinic Telehealth Program for Patients with Uncontrolled Hypertension (2024)	UAB	Adults with hypertension	Clinical/ Telehealth Intervention
	AI-Guided Risk Stratification for Medication Reconciliation for Patients with Diabetes (2025)	UAB	Adults with diabetes	Digital Health
	A Clinical Decision Support Questionnaire to Identify Obstacles to Blood Pressure Control (2025)	UAB	Adults with uncontrolled blood pressure	Clinical Tool Development
	African American Stress Intervention Study: Feasibility in Hypertension (ASIS-Feasibility HTN Cohort; 2022)	NCATSU	African American adults with hypertension	Behavioral/Feasibility
	Feasibility and Acceptability of a Yoga Intervention in Heart Failure Patients with Mild Cognitive Impairment (2025)	AU	Adults with heart failure and cognitive impairment	Behavioral Intervention
	Enhancing Patient-Centered Care for Older African American Patients with Cardiovascular Comorbidities (2025)	UAB	African American adults with cardiovascular disease	Care Delivery Intervention
Social determinants of health & structural interventions	Social Determinants of Health and their Impact on Establishing Heart Failure Care in a Clinic for the Underserved (2022)	UAB	Underserved patients with heart failure	Observational/Health Systems
	Targeting Healthy Weight Loss in the Context of Food Insecurity: Pilot and Feasibility Trial I & II (2022, 2023)	PBRC	Food insecure women with obesity	Behavioral/Nutrition Intervention
	Facilitated Stable Housing as a Strategy for Uptake and Sustainment of Evidence-Based HIV and Cardiometabolic Medicine in People with HIV (2024)	UAB	Adults with HIV	Structural/Implementation
	Impact of a Health Coaching Program to Improve the Health of Food-Insecure Adults with Cardiovascular Disease (2025)	UAB	Adults with food insecurity	Behavioral Intervention
	Pharmacist-Led Remote Patient Monitoring for Diabetes and Hypertension Management in Persistent Poverty Areas (2025)	UAB	Adults with pre-diabetes, diabetes, or hypertension	Telehealth/Care Delivery
	An Integrated Cardiometabolic Intervention Targeting Physical and Financial Health: A Pilot Study (2024)	UAB	Low to moderate income Black adults with cardiometabolic risk factors	Behavioral Intervention
Maternal, reproductive, and women’s health	Teen Mom 2: A Multicomponent Digital Health Intervention to Improve Black Maternal Cardiometabolic Health in Mississippi’s WIC Community (2022)	UMMC	Pregnant Black adolescent WIC clients	Digital Health Intervention
	Use of Home-Delivered Meals to Manage Cardiometabolic Health During Pregnancy (2022)	UAB	Low-income, Black pregnant women	Nutrition Intervention
	Evaluating the Utilization of Remote Patient Monitoring for Hypertensive Disorders of Pregnancy (2025)	UA	Pregnant and postpartum women	Digital Health/Monitoring
	Evaluating the Utilization of the Heart Truth Program Among Young Black Women in the Deep South (2025)	UA	Young black women with hypertension, diabetes, or obesity	Program Evaluation
HIV and comorbid cardiometabolic conditions	Addressing Cardiometabolic Disease in People Living with HIV: Developing Strategies to Improve Diet and Exercise by Assessing Multilevel Social Determinants of Health (2023)	UAB	Adults with HIV	Behavioral/Observational
	Peer MODELS: Managing a Community-Based HIV, Diabetes, and Pain Intervention (2024)	UAB	Adults with HIV and diabetes	Behavioral/Peer Support
	Enhancing Recruitment and Retention for Black Females with HIV: Identifying Barriers, Facilitators, and the Role of Social Determinants for the Willingness to Participate in HEALTH study (2024)	UAB	Black women with HIV	Implementation Science
	Laying the Foundation for PrEP in Urgent Care Settings (2024)	UAB	Urgent care clinic staff and patients	Implementation/Prevention
Community-based & behavioral interventions	Mobile Community Stroke Self-Management Program: A Feasibility Study (2023)	UA	African American stroke survivors in rural Alabama	Behavioral Intervention
	Preliminary Implementation and Development of an Enjoyable Virtual Reality Exercise Program for the Prevention of Cardiometabolic Disease Among Children with Cerebral Palsy in School Settings (2023)	UAB	Children with cerebral palsy and their caregivers, teachers	Digital/Behavioral Intervention
	Empowering Faith-Based Communities to Deliver Personalized Diabetes Self-Management Education and Support (2025)	UAB	Adults with diabetes	Community-Based Intervention
	Assessing the Feasibility and Preliminary Efficacy of a Community Health Worker Program in a Safety Net Clinic (2025)	AU	Adult community health worker trainees	Community-Based Intervention
	Building Trust to Improve Cardiovascular Health Among Black Americans (2025)	UMMC	Black adults at risk of cardiometabolic disease	Community Engagement
Special populations, risk prediction, & precision health	Sickle Cell Fit: Increasing Physical Activity in Youth with Sickle Cell Disease (2023)	UMMC	Children and young adults with sickle cell disease	Behavioral Intervention
	Development and Validation of a Diabetes Risk Score for Undiagnosed Hispanics in the United States (2025)	UAB	Hispanic adults at risk of diabetes	Risk Prediction Model
	Linking Genetics and Improving Nutrition in Scotlandville (2024)	PBRC	African American adults with cardiometabolic risk factors	Community-Based Intervention
Dyadic & family-based interventions	A Feasibility Trial of a Stakeholder-Enhanced, Lay Navigator-Delivered Intervention to Improve the Decisional Partnership of Chronic Kidney Disease Dyads (2023)	UAB	Black adults with stage 3 or 4 chronic kidney disease and their care partners	Behavioral/Care Coordination
	Heart Care Pairs: A Primary Care-Based Dyadic Cardiovascular Risk Reduction Intervention (Pilot and Mixed Methods Study; 2024 and 2025)	UAB	Adults with hypertension and their care partners	Behavioral Intervention

UMMC = University of Mississippi Medical Center; UAB = University of Alabama at Birmingham; NCATSU = North Carolina Agricultural and Technical State University; AU = Auburn University; PBRC = Pennington Biomedical Research Center; UA = University of Alabama.

**Table 4.** Evaluation metrics by type and center component Table 4 long description.

Center component	Metric type	Evaluation metric
Investigator development core	Process	Number of letters of intent received
		Number of letters of intent received by regional institutions
		Characteristics of investigators submitting letters of intent (e.g., career stage, institution)
		Number of pilot proposals received
		Number of funded pilot proposals
		Characteristics of pilot awardees
		Number of regional institutions represented by funded pilots
	Scholarship	Number of peer-reviewed manuscripts by pilot awardees
		Number of regional/national presentations by Center members
		Number of regional/national presentations by pilot awardees
		Number of extramural grant applications submitted by pilot awardees
		Number of extramural grant applications awarded to pilot awardees
	Societal impact	Number of selected activities implemented and tracked by pilot awardees
		Nonacademic partners’ experience and level of engagement in research projects (as measured by annual survey of Coalition members)
Research projects	Process	Number of projects achieving pre-specified milestones (e.g., recruitment, data collection, retention, analyses)
		Use of common data elements
	Scholarship	Number of peer-reviewed publications
		Number of regional, national, and international presentations
		Number of submitted extramural grant applications
		Number of funded extramural grant applications
	Societal impact	Number of presentations of study findings to health system leadership
		Adoption/implementation of new digital tools or telehealth programs
		Changes to health system screening, referral, or treatment procedures
		Evidence of intervention cost-effectiveness
		Number of health education resources developed for community organizations
		Number of community improvements to built environments
		Number of meetings with local payers, public policy representatives, or legislators to present study findings

## Challenges and lessons learned

Several key lessons have emerged during the implementation of the Pilot Plus program. First, project timelines were highly affected by factors beyond Scholars’ control, including delays in institutional review board approval, difficulties accessing study participants, and the work involved with establishing long-term relationships with community partners to enhance sustainability, underscoring the need for built-in flexibility and extended timelines for early-stage research. Second, recently evolving national funding priorities have highlighted the importance of supporting alternative pathways for scaling up pilot studies when traditional mechanisms are no longer feasible. The program also demonstrated that achieving and sustaining varied institutional and disciplinary participation requires intentional infrastructure, including strong site leadership, identification of institutional champions, and structured opportunities for relationship building beyond virtual engagement. Heterogeneity in institutional resources and research infrastructure to support scholars conducting T3–T4 research revealed the need for strategies that enhance support for pilot awardees conducting pilots in less resourced environments. Finally, the emphasis on community-based participatory research reinforced the value (and inherent complexity) of relationship building, particularly in integrating SDOH and addressing challenges related to scalability and sustainability within the constraints of pilot-level funding.

Although developed within the context of the Deep South, the Pilot Plus model includes several components that are broadly generalizable to other translational science settings. These include the integration of pilot funding with required, cohort-based training; the inclusion of community stakeholders in proposal review processes; the use of multidisciplinary project management teams to support both project execution and career development; and centralized access to methodological expertise across institutions. These elements may be particularly valuable in settings seeking to build translational research capacity across institutions with heterogeneous resources. In contrast, the specific regional partnerships, institutional configurations, and cardiometabolic disease focus of the Forge AHEAD Center are context-specific and may require adaptation to local needs.

In summary, the Forge AHEAD Center has developed and implemented the Pilot Plus model, an integrated approach to workforce development that combines pilot funding with structured training in T3–T4 translational science, multidisciplinary team-based mentorship, and embedded community engagement. By aligning these components within a coordinated framework, the program addresses fragmentation commonly observed in traditional pilot funding and training mechanisms. The program’s emphasis on centralized methodological support and cross-institutional collaboration further enhances access to critical research infrastructure, particularly for investigators in resource-variable settings. While longer-term outcomes related to extramural funding are still being evaluated, this work establishes the foundational infrastructure and approach for supporting ESIs in translational research. Collectively, this approach provides a scalable and adaptable framework for developing a demographically varied translational science workforce equipped to design, implement, and disseminate interventions that address complex health disparities.

## Table 1. Long description

A table with two columns: Criterion and Description. The table has nine rows, each detailing specific criteria for reviewing pilot projects. Row 1: Criterion, Research theme; Description, Applications should clearly describe how the proposed research aligns with the mission of the Center. Row 2: Criterion, Approach; Description, Methods must reflect translational research principles, including implementation/dissemination of effective interventions or program evaluation for efficacy and improvement. Row 3: Criterion, Scientific merit; Description, Evaluated based on NIH criteria: significance, innovation, approach, rigor and reproducibility, and potential for broader implementation or dissemination. Row 4: Criterion, Investigative team; Description, Assessment of the Principal Investigator’s training, demonstrated interest in translational (i.e., T3–T4) research, and potential for independence. Co-investigators are evaluated for necessary expertise and complementary skills. Row 5: Criterion, Environment; Description, The environment must support translational research, including details on academic as well as community or clinical settings relevant to implementation. Row 6: Criterion, Timeline; Description, Applications must justify feasibility of project completion within 1 year or provide rationale for a 2-year project period. Row 7: Criterion, Protection of human subjects; Description, A clear plan for human subjects protections must be provided, including any relevant prior IRB documentation. Row 8: Criterion, Integration with center cores; Description, Proposals must specify which Center Cores will be used and how they will support the project. Row 9: Criterion, Budget; Description, Budget and justification are reviewed for feasibility, appropriateness, and completeness.

Navigate back to Table 1..

## Table 2. Long description

A table with five rows and four columns detailing the components, structure, key activities, primary functions, and unique contributions of a pilot plus curriculum. The columns are labeled Component, Structure/frequency, Key activities, Primary functions, and Unique contributions (Plus). The rows describe Monthly seminars & enrichment series, Project management teams, Community-engaged research integration, Annual scholars meeting, and Specialized support units (translational design, SDOH, communications). Each row provides specific details about the structure, activities, functions, and unique contributions of each component.

Navigate back to Table 2..

## Table 3. Long description

Panel A: Table with columns for thematic area, project title and funding year, institution, target population, and study type/approach. Panel B: Table with columns for thematic area, project title and funding year, institution, target population, and study type/approach.

Navigate back to Table 3..

## Table 4. Long description

A table with 11 rows and 3 columns. The columns are labeled Center component, Metric type, and Evaluation metric. The table is divided into two main sections: Investigator development core and Research projects. Each section is further divided into subcategories: Process, Scholarship, and Societal impact. The Investigator development core section includes metrics such as the number of letters of intent received, characteristics of investigators submitting letters of intent, number of pilot proposals received, number of funded pilot proposals, characteristics of pilot awardees, number of regional institutions represented by funded pilots, number of peer-reviewed manuscripts by pilot awardees, number of regional/national presentations by Center members and pilot awardees, number of extramural grant applications submitted and awarded to pilot awardees, and number of selected activities implemented and tracked by pilot awardees. The Research projects section includes metrics such as the number of projects achieving pre-specified milestones, use of common data elements, number of peer-reviewed publications, number of regional, national, and international presentations, number of submitted and funded extramural grant applications, number of presentations of study findings to health system leadership, adoption/implementation of new digital tools or telehealth programs, changes to health system screening, referral, or treatment procedures, evidence of intervention cost-effectiveness, number of health education resources developed for community organizations, number of community improvements to built environments, and number of meetings with local payers, public policy representatives, or legislators to present study findings.

Navigate back to Table 4..
